# Knowledge, Attitude, and Practice (KAP) toward Cervical Cancer Screening among Adama Science and Technology University Female Students, Ethiopia

**DOI:** 10.1155/2022/2490327

**Published:** 2022-01-13

**Authors:** Almaz Tadesse, Mesfin Tafa Segni, Hailu Fekadu Demissie

**Affiliations:** Department of Public Health, College of Health Science, Arsi University, Asella, Ethiopia

## Abstract

**Background:**

Cervical cancer is a complication of Human Papillomavirus (HPV) infection is the second most common cancer in women worldwide. Eighty percent of the cases occur in low-resource countries. According to the 2009 World Health Organization report, the age-adjusted incidence rate of cervical cancer in Ethiopia was 35.9 per 100,000 patients with 7619 annual number of new cases and 60-81 deaths every year. The study is aimed at assessing the level of knowledge, attitude, and practice concerning cervical cancer among female students at Adama Science and Technology University. *Methodology*. An institutional based cross-sectional study was conducted among 667Adama Science and Technology University female students. A simple random sampling method was used to select the respondents. Structured self-administered questionnaire was used for data collection.

**Results:**

About 404 (60.6%) of the participants heard about cervical cancer, 478 (71.7%) had positive attitude towards cervical cancer screening, and only 15 (2.2%) participants were screened for cervical cancer. Lack of information about cervical cancer was the most reported reason for not attending to cervical cancer screening. *Conclusion and Recommendation*. The study showed that there was low knowledge on cervical cancer and screening for premalignant lesion among women. There is a need to promote and encourage women to early cervical cancer screening at precancerous stage by informing their susceptibility to cervical cancer.

## 1. Introduction

Worldwide, cervical cancer is the second most common health problem in women. Approximately 80% of cervical cancer occur in developing countries [1, 2]. According to Cervical Cancer Crisis Card 2013, cervical cancer kills an estimated 275,000 women every year and 500,000 new cases are reported worldwide. Africa is the most affected region with the highest rate. The highest incidence rates in the world were reported in eastern, western, and southern Africa. Projections show that by 2030, almost half a million women will die of cervical cancer, with over 98% of these deaths expected to occur in low- and middle-income countries [3]. Pap smear (colposcopy), visual inspection with acetic acid (VIA), and HPV testing can help diagnose early cancerous cells [4].

Ethiopia ranked the 20^th^ next to Japan with a mortality rate of 14 per 100,000 with a total death of 3,235 due to cervical cancer in 2013 [3, 5]. The age-adjusted incidence rate of cervical cancer in Ethiopia was 35.9 per 100,000 women with an annual number of 7,619 new cases and 60-81 deaths every year [6]. One major determinant for the prognosis of cervical cancer is the stage at which the patient presents [7]. Most patients in developing countries including Ethiopia present late with advanced stage disease, in which treatment may often involve multiple modalities including surgery, radiotherapy, and chemotherapy and has a markedly diminished chance of success [8]. Several factors such as educational status, financial capability, location, and presence of health care facilities determine the stage at which patients with cancer present to the health facility. However, the common denominator of these factors is the level of awareness and attitude patients have about the diseases [5].

While numerous tools and technologies exist to prevent cervical cancer, these interventions remain largely inaccessible to girls and women who live in a developing country. Despite the proven link between the Human Papillomavirus (HPV) and cervical cancer, HPV vaccines not widely available and screening rates remain low in a developing country, especially in Sub-Saharan Africa. Lack of awareness and deep-seated stigma associated with the disease also pose significant barriers to access [3].

Over the years, the Ethiopian Federal Ministry of Health has been trying to deal with this problem by providing resources at its Family Guidance Clinics as well as the laboratories and training its staffs especially the staff nurses to be certified in conducting Pap smear screening to reduce the incidence [9]. Now, the government has been trying to expand the service to all levels by training staffs in 10-day trainings to conduct screening using visual inspection with acetic acid (VIA) to all women that need the services freely. Despite this fact, very few women receive screening services due to different factors like awareness problem and shortage of supply in Ethiopia.

Although there is no national cancer registry in Ethiopia, reports from a retrospective review of biopsy results have shown that cervical cancer is the most prevalent cancer among women in the country followed by breast cancer [10]. Low level of awareness, lack of effective screening programs, overshadowed by other health priorities (such as AIDS, TB, and malaria), and insufficient attention to women's health are the possible factors for the observed higher incidence rate of cervical cancer in the country [11].

Data on knowledge of Ethiopian female college students regarding cervical cancer is scarce. This study is aimed at assessing knowledge, attitude, and practice (KAP) and associated factors of cervical cancer among female college students at Adama University. So the finding of this study will help to develop ways to improve the KAP towards cervical cancer among female students and other women in the community in Ethiopia and elsewhere for similar community.

## 2. Methods

### 2.1. Study Design, Setting, and Population

An institutional based cross-sectional study design was conducted at Adama Science and Technology University in its two campuses found in Adama and Asella towns located 100 and 175 kilometres, respectively, from Addis Ababa toward East. The study was conducted in May 2014 among regular undergraduate female students who were registered for the academic year of 2013/2014. Those who were ill and absent, with extension, summer, and distance education, and postgraduate students were excluded from this study.

### 2.2. Sample Size and Sampling Procedures

Sample size was calculated using the single proportion formula.

(1) $$\begin{array}{c} n=\frac{{\left(Z_{\alpha /2}\right)}^{2}\;P\left(1-P\right)\;}{d^{2}}, \end{array}$$

where *Z* is the 95% confidence interval (1.96), *d* is the marginal error (3%), *n* is the sample size, *P* is the estimated proportion, and *Z*_*α*/2_is the critical value.

From a study done on KAP of cervical cancer among reproductive health clients at three teaching hospitals in Addis Ababa, the prevalence of knowledge was 19% [9]. The required sample size was 690.

Multi stage sampling technique was used to select the respondents of the study. First, one department was selected from seven schools of Adama Science and Technology University; by using simple random sampling, the sample size was proportionally allocated for each selected department based on their class size. Secondly, using the student list obtained from the office of registrar as a sampling frame, the respondents were selected by a simple random sampling method.

### 2.3. Data Collection Procedures and Assurance

A self-administered close-ended structured questionnaire developed from different literatures was used for collecting the data which was prepared in English and then translated to the national language Amharic. Training was given for two supervisors and four field workers on the purpose of study and procedures of data collection for two days prior to the study. A pretest was done among 5% of the total sampled population not included in the study, and amendment was made on the questionnaires. Instructors who had class at the time of data collection were informed about the study through telephone, and a letter of support from the department for permission was provided before beginning the class. The identification number of the selected students in the study was posted in the class. Then, the data collectors described to the students about the objective of the study and administered the questionnaire by crosschecking their identification number. Finally, the field workers collected the filled questionnaires and supervisors cross checked the completeness of the questionnaires.

### 2.4. Outcome Measurement

#### 2.4.1. Knowledge Assessment

The knowledge of the cervix cancer and screening for premalignant cervical lesion was assessed using an 11-point scale. There were eight multiple choice questions that carried a total of 11 correct responses. Each correct response was given a score of 1 and a wrong response a score of 0. Total points to be scored were 11, and the minimum was 0.

Using Bloom's cutoff points for KAP study, a score of 80–100% of correct responses meant a good knowledge, a score of 50–79% puts a scorer in a level of satisfactory knowledge, and a poor knowledge was for the respondents with a score less than 50% of the correct responses.

#### 2.4.2. Attitude Assessment

Attitude was assessed using 7 Likert scale items with responses: strongly agree, agree, neither agree nor disagree, disagree, and strongly disagree. The scoring system used was as follows: 5—strongly agree, 4—agree, 3—neutral, 2—disagree, and 1—strongly disagree. The highest score was expected to be 35 and the lowest score to be 7 for each respondent. The mean score was calculated, and those who scored above the mean had positive attitude, and those who scored below the mean had negative attitude towards screening for premalignant cervical lesions.

#### 2.4.3. Practice Assessment

The practice was assessed by looking on the respondent's action towards screening for premalignant cervical lesion in the past three years. Those who were screened within the past three years were regarded as having regular practice, and those who were never screened were regarded as having no practice on screening.

### 2.5. Data Handling and Analysis

The collected data were entered into SPSS version 21 for analysis. After the entrance and completeness of all data, cleaning was done. Descriptive analysis using frequency, mean, median, standard deviation, and percentages was done. A chi-square test was used to assess association between knowledge and attitude and knowledge and practice. Binary logistic regression was used to assess relationship between independent variables with outcome variables to control confounding effect and to determine adjusted odds ratio (AOR). The variables included in the multivariate logistic regression were variables produced with a *p* value of ≤0.3 on the bivariate analysis. The results of the final model were expressed in terms of odds ratio (OR) and 95% confidence intervals (CI) and statistical significance was declared if the *p* value is less than 0.05.

### 2.6. Ethics Statement

Ethical clearance was obtained from Research and Ethics Committee (REC) of School of Public Health, Addis Ababa University. Oral consent was obtained explaining the purpose of study, the right to refuse participation or to jump some questions unwilling to answer. To ensure the confidentiality of respondents, their names were not written on the questionnaire.

## 3. Result

### 3.1. Sociodemographic Characteristics of Respondents

A total of 667 students completed the questionnaire making the response rate of 97%. Majority (568, 85.2%) of the respondents were younger than 30 years old. Majority (497, 74.5%) of the respondents were from urban areas. Regarding year of education, 258 (38.7%) were first year students and 214 (32.1%) were second year students. About 408 (61.3%) were Orthodox Christian religion followers (Table 1).

### 3.2. Knowledge of Respondents on Cervical Cancer

Concerning the overall knowledge score, 14.8% of the respondents had good knowledge. Majority (404, 60.6%) of the students heard about cervical cancer. Of these who had heard about cervical cancer; the most frequent source of information was mass media (232, 57.4%) and the least source of information (7, 1.7%) was from religious leaders (Table 2).

Less than one-third (211, 31.6%) and more than one-fifth (147, 22.0%) of the respondents mentioned vaginal foul smelling discharge and vaginal bleeding during sexual intercourse as the symptom of cervical cancer. When asked on the knowledge about the symptoms and risk factors for cervical cancer, 270 (40.5%) respondents revealed to be having multiple partners and 197 (29.5%) to be having Human Papillomavirus (HPV) (Table 3).

Table 4 shows cervical cancer prevention, treatment, and screening options. More than half (354, 53.1%) of the participants knew that cervical cancer is prevented by avoiding multiple sexual partners. Regarding the treatment, 319 (47.8%) participants knew that cervical cancer is treatable, 303 (45.4%) do not know whether it is treatable or not, and 45 (6.7%) participants said cervical cancer cannot be treated.

Concerning how frequent one should be screened for cervical cancer, majority (431, 64.6%) did not respond on the frequency of screening. Majority (409, 61.3) of the respondents answered that women of above 25 years of age should be screened. Two hundred twenty seven (34.0%) participants knew that biopsy is used as one method of screening procedures of cervical cancer, 159 (23.8%) Pap smear, and 57 (8.5%) VIA (Table 4).

### 3.3. Association between Sociodemographic Characteristics and Knowledge Score

In both bivariate and multivariate analyses, place of birth and level of education were significantly associated with the knowledge score of the respondents. Students who were born at urban areas were more than two times knowledgeable than those born at rural area (AOR = 2.64, 95% CI: 1.46, 4.75). Year three female students were more than two times more knowledgeable about cervical cancer (AOR = 2.21, 95% CI: 1.25, 3.90). Year four and above female students were about four times more knowledgeable than the 1^st^ and 2nd year students (AOR = 3.92, 95% CI: 2.08, 7.40) (Table 5).

### 3.4. Attitude of Respondent about Screening of Cervical Cancer

As shown in Table 6, 478 (71.7%) of the respondents agreed that carcinoma of the cervix causes death. Two-thirds (66.4%) of the respondents perceived that any woman can acquire cervical cancer, 488 (73.2%) of the respondents agreed that screening helps in the prevention of cervical cancer. If screening of cervical cancer is free and the procedure cannot cause any harm, 459 (68.9%) respondents will have positive attitude to be screened.

### 3.5. Association Factor toward Attitude of Cervical Cancer Screening

Sexual experience, place of birth, and level of education were associated with positive attitude towards cervical cancer screening in the bivariate analysis. But in multivariate analysis, sexual experience and level of education remained significantly associated with positive attitude. Previous sexual experience increases the odds of cervical cancer screening uptake by two times (AOR = 1.87, 95% CI: 1.32, 2.64). First year female students had more positive perception toward cervical cancer screening than senior female students (AOR = 2.09, 95% CI: 1.18, 3.69) (Table 7).

### 3.6. Practice towards Screening for Cervical Cancer

Only 2.2% of the respondents were screened in their lifetime. When asked reasons for not being screened, 283 (42.4%) said that they did not have information, 187 (28.0%) said they are healthy, 99 (14.8%) have not decided to be screened, 37 (5.5%) feel shy, and 17 (2.5%) said screening was expensive (Table 8).

None of the sociodemographic characteristics were significantly associated with practice of cervical cancer screening.

## 4. Discussion

In this study knowledge, attitude and practice about cervical cancer screening were examined. In this study, three-fifth (60.6%) of the participants heard about cervical cancer. This finding is lower than that of the study in Spain in 2014 [12] and Nigeria in 2014 [13], but higher than the finding in Zimbabwe, with only 90% of the respondents having not heard about cervical cancer [14]. The main source of information was mass media which agrees with a study done in Hong Kong in 2014 [15]. This indicates that media can play an important role in educating women regarding cervical cancer. Medias like radio and TV are nowadays accessible in many households where information can simply reach to the wide community without any additional cost. Contrary to this, a study done in Kenya reported that the main sources of information were health care providers [16], and in Addis Ababa 2008, the main source of information was health institutions [9]. The difference could be due to the study subjects and the study place; the respondents were reproductive health clients in the previous studies.

The overall good knowledge score of the respondents was 14.8%. These results indicate that information of cervical cancer screening was inadequate among our study group. It might therefore contribute to delaying of establishment of prevention and screening effort community. Similar low level findings were reported in a study in Nigeria in 2013 [17]. The finding is very much lower than study finding in Indian, Nepal, and Yemen [18–20]. About one-third of the participants knew that vaginal foul smelling discharge is a symptom of cervical cancer. This difference may be due to cultural and socioeconomic deference among these two populations.

Having multiple sexual partners was the major risk factor reported for cervical cancer, followed by acquiring HPV, early sexual intercourse, and cigarette smoking. Studies in German 2005 and Uganda in 2006 and 2013 confirm this finding. HPV infection, multiple sexual partner, early sexual initiation, and smoking were reported as risk factors for developing cervical cancer [21, 22].

Concerning prevention of cervical cancer, over a half of the participants knew that cervical cancer is prevented by avoiding multiple sexual partners, avoiding early sexual intercourse, and quitting smoking while in a study done in Spain, 67% of cervical cancer can be prevented by early screening and HPV vaccination [12]. This disparity may be due to the developed nations like Sweden; early screening and availability of HPV vaccination could be affordable for most of the population at every facility.

In multivariate logistic regression, place of birth and academic year of the students were significantly associated with good knowledge score. Females who were born at urban areas were more likely to be knowledgeable than those born at rural areas. Mostly, respondents from urban are nearest to different health-related information because of accessibility to multimedia. Year three female students were more than two times knowledgeable about cervical cancer. Year four and above female students were about four times more knowledgeable than the 1^st^ and 2^nd^ year students. This may be as academic year and the length of stay in campus increase; they might be introduced about cervical cancer or participation in different clubs like HIV AIDS and help to create awareness among the students. For medical students, they definitely are aware in their major courses like Surgery and Gynecology.

About two-thirds of the respondents perceived that any woman can acquire cervical cancer. Nearly three-quarters (73.2%) of the respondents agreed that screening helps in the prevention of cervical cancer. Two hundred ninety seventy (44.5%) had positive attitude, and the remaining 370 (55.5%) had negative attitude towards cervical cancer screening. In multivariate logistic regression, previous sexual experience and academic year of the students were significantly associated willingness to screening. Sexually active females can get information regarding cervical cancer from health professionals or their friends and by going to health facility to get services for reproductive health issues like family planning, menstrual problems, and STI. They may have been counseled or advised by health care providers regarding cervical problems. First year female students had more positive perception towards cervical cancer screening than senior female students.

This study showed that more than two-thirds of the respondent had a positive attitude towards screening of cervical cancer while a study done in the UK showed majority of women had negative attitude [23]. In some studies, Latinos and women of Asian descent endorsed more misconceptions about cancer and fatalistic beliefs [24]. This is much higher than a study done in Songea, Ruvuma: on knowledge, attitude, practice, and perceived barriers towards screening for premalignant cervical lesions among women aged 18 years and above were 18%. But it is lower than the finding in a study in Nepal (85%, 19) and Zimbabwe (80%, 14) where majority of the respondents had positive attitude toward cervical cancer screening.

The practice of cervical cancer screening among participants of this study is very much low (2.2%), compared to studies done in Spain (71.5%, 12), in Hong Kong (7%, 15), in Nepal (10.5%, 19), in Yemen (7%, 20), in Nigeria (6%) [4], in Uganda (19%) [22], and in Addis Ababa (6.5%) [9]. The main reasons mentioned for not being screened were lack of information, absence of symptoms (being healthy), or not deciding to be screened. Similarly in Nigeria, the main reasons for not being screened were lack of awareness, dislike of pelvic examination, and absence of symptom [4]. But in a study from Yemen, shyness, thinking of no need to do it, and fear of the procedure were the main reasons for not practicing screening [20]. A study in Uganda also reported reasons like not feeling at risk, lack of symptoms, carelessness, fear of vaginal examinations, lack of interest, and test being unpleasant [22]. The other justification for not being screened could be about 85.2% of the respondents were 20 years old or younger and most did not have a sexual experience.

The study might be subjected to social desirability bias due to self-administering the questionnaires. The other limitation of the study could be due to the nature of the study being cross-sectional. In addition, the age of the data which was conducted in 2014 might be one limitation and further study is recommended with complementation of qualitative study.

## 5. Conclusions

The level of knowledge about cervical cancer was inadequate among the study participants. In addition, the uptake premalignant cervical cancer lesion screening was low. The most reasons for low practice of screening are being health and lack of information. The government should play its part by increasing health care budgets and put priority on cervical cancer prevention by establishing a national awareness campaign, spreading screening services all over the country using cheap screening procedures that have shown to have reasonable sensitivity and specificity.

## Figures and Tables

**Table 1:** Sociodemographic characteristic of respondents for KAP of Cervical Screening at Adama Science and Technology University Adama, Ethiopia, May 2014.

Characteristics	Category	Number	Percent
Age of respondent in years	15-20	568	85.2%
	>20	99	14.8%

Sexual experience	Yes	219	32.8%
	No	448	67.2%

Age at first sex in years	15-20	154	72.3%
	>20	59	27.7%

Number of sexual partner	Single	185	87.3%
	Multiple	27	12.7%

Place of birth	Urban	497	74.5%
	Rural	170	25.5%

Level of education	Year 1	258	38.7%
	Year 2	214	32.1%
	Year 3	125	18.7%
	Year 4 & above	70	10.5%

Religion	Orthodox	408	61.2%
	Catholic	32	4.8%
	Muslim	86	12.9%
	Protestant	128	19.2%
	Other	13	1.9%

**Table 2:** Respondents' knowledge about cervical cancer screening at Adama Science and Technology University, Adama, Ethiopia, May 2014.

Knowledge variable	Category	Number	Percent
Knowledge score	Good	99	14.8%
	Poor	568	85.2

Heard about cervical cancer	Yes	404	60.6%
	No	263	39.4

Source of information (*n* = 404)	Heard from news media	232	57.4%
	Heard from health worker	133	32.9%
	Heard from family, neighbor, friend	78	19.3%
	Heard from teacher	69	17.1%
	Heard from broacher& other	67	16.6%
	Heard from religious leaders	7	1.7%

The sum of percentage is >100 because of multiple responses.

**Table 3:** Respondents' knowledge about symptoms and risk factors of cervical cancer at Adama Science and Technology University students, Adama, Ethiopia, May 2014.

Symptoms of cervical cancer	Category	Frequency	Percent
Vaginal bleeding is symptom of cervical cancer	Yes	147	22.0%
	No	520	78.0%
Vaginal foul smelling is symptom of cervical cancer	Yes	211	31.6%
	No	456	68.4%
*Risk factor for cervical cancer*			
Multiple sexual partner is a risk factor	Yes	270	40.5%
	No	397	59.5%
Early sexual intercourse is a risk	Yes	146	21.9%
	No	521	78.1%
Acquiring HPV is a risk	Yes	197	29.5%
	No	470	70.5%
Cigarette smoking is a risk	Yes	61	9.1%
	No	606	90.9%

**Table 4:** Respondents' knowledge about prevention, treatment, and screening modalities of cervical cancer at Screening at Adama Science and Technology University, Adama, Ethiopia May 2014.

Variable	Category	Frequency	Percent
Prevention methods	Avoiding multiple sexual partners prevents cervical	354	53.1
	Avoiding early sexual intercourse	222	33.3
	Quitting smoking prevents cervical cancer	94	14.1
	HPV vaccination prevents cervical cancer	242	36.3
	Screening prevents cervical cancer	306	45.9

Knowing cancer of the cervix can be treated	Yes	319	47.8
	No	45	6.7
	Do not know	303	45.4

Treatment type (*n* = 319)	Herbal remedies	69	21.6
	Surgery	79	24.8
	Radiotherapy	85	26.6

Frequency of screening	Once a year	176	26.4
	Every three years	38	5.7
	Every five years	11	1.6
	Any other	11	1.6
	Do not know	431	64.6

Who should be screened	Women of >25 years	409	61.3
	Prostitutes	157	23.5
	Elderly women	47	7.0
	Others	54	8.1

Procedures used in cervical cancer screening	VIA	57	8.5
	Pap smear	159	23.8
	Biopsy	227	34.0

**Table 5:** Association between sociodemographic characteristics and knowledge score of cervical cancer among Adama Science and Technology University, Adama, Ethiopia, May 2014.

Variables	Category	Knowledge score		COR (95% CI)	AOR (95% CI)
		Poor	Good		
Sexual experience	No	375 (83.7)	73 (16.3)	1	1
	Yes	176 (80.4)	43 (19.6)	0.80 (0.53, 1.21)	1.23 (0.79, 1.92)

Place of birth	Urban	396 (79.7)	101 (20.3)	2.64 (1.20)	2.64 (1.46, 4.75)
	Rural	155 (91.2)	15 (8.8)	1	1

Level of education	Year 1	227 (88.0)	31 (12.0)	1	1
	Year 2	185 (86.4)	29 (13.6)	1.15 (0.67, 1.97)	1.08 (0.62, 1.87)
	Year 3	94 (75.2)	31 (24.8)	2.42 (1.39, 4.20)	2.21 (1.25, 3.90)
	Year 4 & above	45 (64.3)	25 (35.7)	4.07 (2.20, 7.54)	3.92 (2.08, 7.40)

Religion	Orthodox	330 (80.9)	78 (19.1)	0.53 (0.16, 1.77)	0.60 (0.20, 2.06)
	Catholic	29 (90.6)	3 (9.4)	0.23 (0.04, 1.24)	0.35 (0.06, 2.01)
	Muslim	72 (83.7)	14 (16.3)	0.44 (0.12, 1.62)	0.61 (0.16, 2.40)
	Protestant	111 (86.7)	17 (13.3)	0.35 (0.10, 1.24)	0.37 (0.10, 1.41)
	Other	9 (69.2)	4 (30.8)	1	

**Table 6:** Respondents' attitudes towards cervical cancer screening at Adama Science and Technology University, Adama, Ethiopia, May 2014.

Variable	Category	Frequency	Percent
Carcinoma of the cervix is the cause of death	Agree	478	71.7%
	Neither agree nor disagree	60	9.0%
	Disagree	129	19.3%

Any woman acquires cervical cancer	Agree	443	66.4%
	Neither agree nor disagree	54	8.1%
	Disagree	170	25.5%

Screening helps in the prevention of cervical cancer	Agree	488	73.2%
	Neither agree nor disagree	69	10.3%
	Disagree	110	16.5%

Willingness for screening	Agree	459	68.9%
	Neither agree nor disagree	59	8.8%
	Disagree	149	22.3%

If screening for cancer is free, will you be screened?	Agree	534	80.1%
	Neither agree nor disagree	36	5.4%
	Disagree	97	14.5%

Because of small numbers of responses for strongly agree and strongly disagree, strongly agree merged to agree, and strongly disagree merged to disagree.

**Table 7:** Association of sociodemographic characteristics and attitude towards cervical cancer screening among respondents at Adama Science and Technology University, Adama, Ethiopia, May 2014.

Variables		Attitude		COR (95% CI)	AOR (95% CI)
		Negative	Positive		
Sexual experience	No	227 (50.7)	221 (49.3)	1.83 (1.31, 2.56)^∗∗^	1.87 (1.32, 2.64)^∗∗^
	Yes	143 (65.3)	76 (34.7)	1	1

Place of birth	Urban	304 (57.6)	224 (42.4)	1	1
	Rural	66 (47.7)	73 (52.5)	1.50 (1.03, 2.18)^∗^	1.19 (0.83, 1.71)

Level of education	Year 1	124 (48.1)	134 (51.9)	2.36 (1.35, 4.13)^∗∗^	2.09 (1.18, 3.69)^∗^
	Year 2	117 (54.7)	97 (45.3)	1.81 (1.02, 3.21)^∗^	1.60 (0.90, 2.87)
	Year 3	81 (64.8)	44 (35.2)	1.19 (0.64, 2.21)	1.03 (0.54, 1.94)
	Year 4 & above	48 (68.8)	22 (31.4)	1	1

Religion	Orthodox	232 (56.9)	176 (43.1)	0.65 (0.22, 1.97)	0.52 (0.17, 1.63)
	Catholic	14 (43.8)	18 (56.3)	1.10 (0.30, 4.02)	0.88 (0.23, 3.34)
	Muslim	42 (48.8)	44 (51.2)	0.90 (0.28, 2.89)	0.65 (0.20, 2.18)
	Protestant	76 (59.4)	52 (40.6)	0.59 (0.19, 1.85)	0.49 (0.15, 1.60)
	Other	6 (46.2)	7 (53.8)	1	1

**Table 8:** Respondents' history of screening for cervical cancer at Adama Science and Technology University, Adama, Ethiopia, May 214.

Variable	Category	Frequency	Percent
Have you ever been screened for cervical cancer?	Yes	15	2.2%
	No	652	97.8%

How many times screened (*n* = 15)	Once	14	2.1%
	More than once	1	0.1%

Reason for not being screened (*n* = 652)	It may be painful	22	3.4%
	I feel shy	37	5.7%
	I am healthy	187	28.7%
	My husband would not agree	4	0.6%
	A screening test reveals cancer	3	0.6%
	It is expensive	17	2.6%
	I am not informed	283	43.4%
	I have not decided	99	15.2%

## Data Availability

Additional detailed information and raw data will be shared upon request addressed to the corresponding author.

## References

[1] Lozano R., Naghavi M., Foreman K. (2012). Global and regional mortality from 235 causes of death for 20 age groups in 1990 and 2010: a systematic analysis for the Global Burden of Disease Study 2010. *The Lancet*.

[2] Ferlay J., Shin H. R., Bray F., Forman D., Mathers C., Parkin D. M. (2010). Estimates of worldwide burden of cancer in 2008: GLOBOCAN 2008. *International Journal of Cancer*.

[3] WHO, UN Cervical Cancer Global Crisis Card. http://www.who.int/hpvcentre/statistics/.

[4] Akinwuntan A. L., Adesina O. A., Okolo C. A. (2008). Correlation of cervical cytology and visual inspection with acetic acid in HIV-positive women. *Journal of Obstetrics and Gynaecology*.

[5] Yusufu L. M. D. (2004). Early diagnosis of breast cancer. *Annals of African Medicine*.

[6] WHO/ICO (2009). *Human Papilloma Virus and Related Cancers in Ethiopia*.

[7] Anima J. T. (1993). Breast cancer in sub-Saharan African women. *African Journal of Medicine and Medical Sciences*.

[8] Wittet S., Tsu V. (2008). Cervical cancer prevention and the millennium development goals. *Bulletin of the World Health Organization*.

[9] Terefe Y., Gaym A. (2008). Knowledge, attitude and practice of screening for carcinoma of the cervix among reproductive health clients at three teaching hospitals, Addis Ababa, Ethiopia. *Ethiopian Journal of Reproductive Health*.

[10] Bekele L. (2009). *Evaluation of Serological Response to Oncoproteins of Human Papiloma Virus Type 16 and 18 as Potential Sero Markers for Cervical Cancer Screening*.

[11] Waktola E. A., Mihret W., Bekele L. (2005). HPV and burden of cervical cancer in East Africa. *Gynecologic Oncology*.

[12] Navarro-Illana P., Diez-Domingo J., Navarro-Illana E., Tuells J., Alemán S., Puig-Barberá J. (2014). Knowledge and attitudes of Spanish adolescent girls towards human papillomavirus infection: where to intervene to improve vaccination coverage. *BMC Public Health*.

[13] Wright K. O., Aiyedehin O., Akinyinka M. R., Ilozumba O. (2014). Cervical cancer: community perception and preventive practices in an urban neighborhood of Lagos (Nigeria). *International Scholarly Research Notices*.

[14] Mupepi S. C., Sampselle C. M., Johnson T. R. (2011). knowledge, attitudes, and demographic factors influencing cervical cancer screening behavior of Zimbabwean women. *Journal of Women’s health*.

[15] Lee A., Ho M., Cheung C. K. M., Keung V. M. W. (2014). Factors influencing adolescent girls’ decision in initiation for human papillomavirus vaccination: a cross-sectional study in Hong Kong. *BMC Public Health*.

[16] Gichangi P., Estambale B., Bwayo J. (2003). Knowledge and practice about cervical cancer and Pap smear testing among patients at Kenyatta National Hospital, Nairobi, Kenya. *International Journal of Gynecological Cancer Supplement*.

[17] Owoeye I. O. G., Ibrahim I. A. (2013). Knowledge and attitude towards cervical cancer screening among female students and staff in a tertiary institution in the Niger Delta. *Int J Med Biomed Res*.

[18] Naik P. R., Nagaraj K., Nirgude A. S. (2012). Awareness of cervical cancer and effectiveness of educational intervention programme among nursing students in a rural area of Andhra Pradesh. *Healthline*.

[19] Shrestha J., Saha R., Tripathi N. (2013). Knowledge, attitude and practice regarding cervical cancer screening amongst women visiting Tertiary Centre in Kathmandu, Nepal. *Nepal Journal of Medical Sciences*.

[20] Abdul-Aziz M. (2012). Knowledge, attitude and practice towards cervical cancer among reproductive health clients at the University of Science & Technology Hospital-Sana'a in Yemen. *Yemeni Journal for Medical Sciences*.

[21] Klug S. J., Hetzer M., Blettner M. (2005). Screening for breast and cervical cancer in a large German city: participation, motivation and knowledge of risk factors. *The European Journal of Public Health*.

[22] Mutyaba T., Mmiro F. A., Weiderpass E. (2006). Knowledge, attitudes and practices on cervical cancer screening among the medical workers of Mulago Hospital, Uganda. *BMC Medical Education*.

[23] Fylan F. (1998). Screening for cervical cancer: a review of women’s attitudes, knowledge and behavior. *British Journal of General Practice*.

[24] Nelson K., Geiger A. M., Carol M. (2002). Effect of health beliefs on delays in care for abnormal cervical cytology in a multiethnic population. *Journal of General Internal Medicine*.

